# Toxo: a library for calculating penetrance tables of high-order epistasis models

**DOI:** 10.1186/s12859-020-3456-3

**Published:** 2020-04-09

**Authors:** Christian Ponte-Fernández, Jorge González-Domínguez, Antonio Carvajal-Rodríguez, María J. Martín

**Affiliations:** 1 Universidade da Coruña, CITIC, Computer Architecture Group, Facultad de Informática, A Coruña, 15071 Spain; 2 Departamento de Bioquímica, Genética e Inmunología y Centro de Investigación Mariña (CIMUVIGO), Universidad de Vigo, Vigo, 36310 Spain

**Keywords:** Simulation, Epistasis model, Gene interaction, Penetrance, Prevalence, Heritability

## Abstract

**Background:**

Epistasis is defined as the interaction between different genes when expressing a specific phenotype. The most common way to characterize an epistatic relationship is using a penetrance table, which contains the probability of expressing the phenotype under study given a particular allele combination. Available simulators can only create penetrance tables for well-known epistasis models involving a small number of genes and under a large number of limitations.

**Results:**

Toxo is a MATLAB library designed to calculate penetrance tables of epistasis models of any interaction order which resemble real data more closely. The user specifies the desired heritability (or prevalence) and the program maximizes the table’s prevalence (or heritability) according to the input epistatic model boundaries.

**Conclusions:**

Toxo extends the capabilities of existing simulators that define epistasis using penetrance tables. These tables can be directly used as input for software simulators such as GAMETES so that they are able to generate data samples with larger interactions and more realistic prevalences/heritabilities.

## Background

The interaction among different genes when expressing a specific phenotype is called epistasis. Its importance in phenotype-genotype associations is well established [1], but traditional GWASs (Genome-Wide Association Study) have only focused on single gene importance or pairwise interactions. However, more recent studies have shown that high-order interactions, those in which more than two loci are involved, may be behind complex traits [2–6].

Epistasis can be defined from different perspectives [1]. Here we focus on statistical epistasis, which refers to the departure from additivity when mapping multilocus genotypes to phenotypic variation. In this context, data set simulations are essential for studying and developing new algorithms or methods for epistasis detection. Simulations offer a controlled environment for testing the accuracy of new methods where the expected results are known beforehand. In contrast, real world data are more costly to acquire and provide no direct way of knowing which result is correct.

The most common way to characterize an epistatic relationship is using a penetrance table, one that contains the probability of expressing the phenotype given each particular allele combination. Although it is quite common for simulators to use them, not all of them allow us to generate the penetrance table. SimuPOP [7], HapSample [8], or SBVB [9], for example, can simulate synthetic data sets employing penetrance tables, but they cannot create them.

Three general approaches are used to create the penetrance tables. The first and most simple approach consists in using an epistasis model. Epistasis models are mathematical relationships that define the penetrance value for each genotype combination as a function of one or more variables, each one usually representing a statistical parameter of the interaction. We can take as examples the well-known models proposed by Marchini et al. in [10]. In these models, the parameters are the baseline effect (*α*), the genetic effect present at every locus independently of the actual allele combination, and the genotypic effect (*θ*), the increase in the odds of the disease beyond the baseline level due to genetic interaction. From these models, a penetrance table can be obtained by giving values to every parameter. However, since penetrances are probability values, they can only take values inside the interval [0,1] and, therefore, there are some restrictions on how the parameter values can be combined. An example of the usage of epistasis models to generate penetrance tables as described can be found in [11].

The second approach is to impose a set of characteristics that should be fulfilled by the simulated population under study and find a penetrance table that complies with these requirements. Parameters model certain characteristics of the population, and the most common are the prevalence *P*(*D*) (representing the proportion of individuals in a population carrying the phenotype of study) and the heritability *h*^2^ (representing the amount of phenotypic variation that corresponds to genetic variation). Finding a table with such requirements is a more complex process than using an epistatic model, therefore a software tool is needed. In this regard, GAMETES [12] is an epistasis simulation software that uses a stochastic method to find a penetrance table with the desired prevalence and heritability levels. It is also able to generate population samples from these tables. GenomeSIMLA [13] is another simulator capable of finding a penetrance table under prevalence and heritability constraints. In this case, it uses a genetic algorithm to reach a solution.

The third and last approach consists in combining the two previous methods: the use of epistasis models together with a set of parametric restrictions. This approach has the advantage of modeling the interaction using the model variables, while also modeling some population characteristics using the parametric restrictions. Consequently, finding a penetrance table is a significantly more complex task. EpiSIM [14] and gs [15] are simulators that fall into this hybrid approach. gs offers the ability to create penetrance tables for nine embedded second-order models, based on the genotype odds ratio(s) for each locus and the prevalence of the desired phenotype. The usability of gs is especially limited due to its restricted set of models. EpiSIM, on the other hand, can create penetrance tables of up to fourth-order and simulate population samples from them. It allows us to specify penetrance values as a function of two variables (i.e., it uses *bivariate* penetrance functions) and it also permtis specifying the desired values of prevalence and heritability. The EpiSIM implementation attempts to find a value for the model variables by solving the equation system made of the prevalence and heritability expressions, respectively defined as:

1 $$ P(D) = {\sum_{i}} P(D|g_{i}) P(g_{i})   $$

2 $$ h^{2} = \frac{{\sum_{i}} \big(P(D|g_{i}) - P(D)\big)^{2} P(g_{i})} {P(D) \big(1 - P(D)\big)}   $$

where *P*(*D*|*g*_*i*_)=*f*_*i*_(*x*,*y*) is the proportion of individuals showing trait *D* when having the genotype *g*_*i*_, *P*(*g*_*i*_) is the population frequency of the genotype *g*_*i*_ and *f*_*i*_(*x*,*y*) is the function of two variables that defines the epistasis model. EpiSIM seeks to find the penetrance table or tables that meet certain prevalence and heritability constraints by solving an equation system made of the two previous expressions. This results in a system with two equations and two unknowns: the two variables of the epistasis model (*x* and *y*).

Although this approach can work for second-order models and low prevalence and heritability values, EpiSIM can barely find solutions to higher-order models or more realistic parameter values. In this paper we present Toxo, a MATLAB library for calculating penetrance tables from models containing bivariate penetrance functions with no limitation on the interaction order. Toxo allows the user to create penetrance tables for a specified epistasis model maximizing the prevalence or heritability when one of the two is constrained. These tables can be used by other simulation packages to generate the data set with the embedded epistasis model and the parametric restriction specified.

## Implementation

### Overview of toxo

Toxo is a MATLAB library designed for calculating penetrance tables using epistasis models containing bivariate penetrance functions, maximizing the prevalence or heritability when one of the two is set. It finds the combination of the two variables from the model that results in a penetrance table where the prevalence is maximum if the heritability was constrained, or the heritability is maximum if the prevalence was the constraint. Toxo does not generate population samples from the tables; instead, it relies on other programs, such as GAMETES [12], to simulate the samples using these tables.

**Fig. 1:**
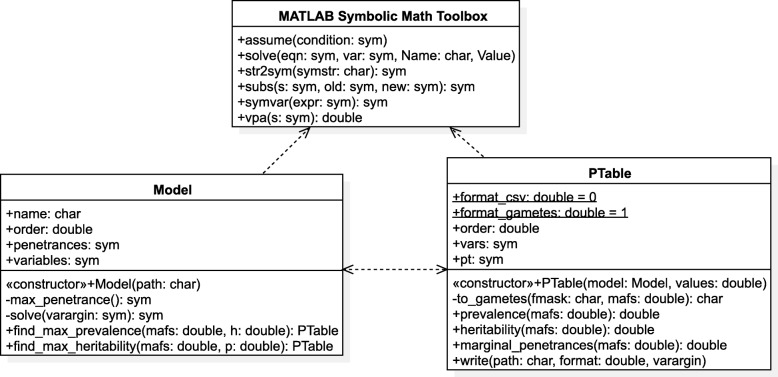
Class diagram of Toxo, representing its two classes Model and PTable, as well as all their attributes and methods. Class Model represents an epistasis model containing bivariate penetrance expressions and offers methods for calculating penetrance tables according to its definition. Class PTable represents a penetrance table and offers methods for calculating parameters from the table and writing it into a file. Both classes use functions provided in the MATLAB Symbolic Math Toolbox

The library consists of two classes, Model and PTable, which encapsulate all the functionality, as represented in Fig. 1. Model class constructor reads the model (provided as a text file) and creates an object representing it. The Model instance offers methods for calculating the penetrance table with the maximum heritability for a certain prevalence, or the table with the maximum prevalence for the specified heritability. These methods return instances of PTable, representing the calculated penetrance table and offering methods for, among other things, writing the table to a file using different formats. In the event of not finding a penetrance table with the desired characteristics, an exception will be raised.

Toxo uses the Symbolic Math Toolbox of MATLAB [16] to represent the models and to calculate the resulting penetrance table. This allows the user to control the precision of the results by changing the precision on all the operations computed within Toxo. If the target prevalence or heritability is a number close to 0 or 1 (the minimum and maximum values, respectively), it may be necessary to increase the number of digits to reduce the error in precision (using the MATLAB function digits).

### Calculating the penetrance tables

**Fig. 2:**
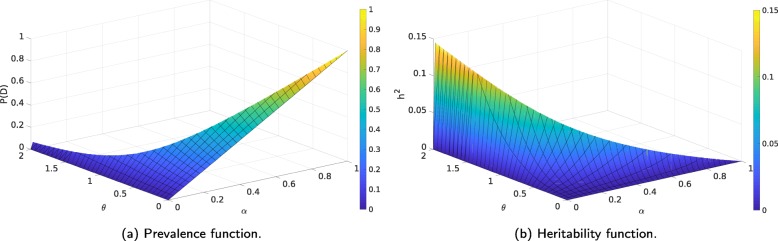
Prevalence and heritability as functions of *α* and *θ* for the second-order additive model shown in Table 1, using *MAF*=0.25, *α*∈[0,1] and *θ*∈[0,2]. Note that prevalence values closer to 0 and heritability values higher than 0.15 can be achieved for values of *θ* higher than two, outside of the area represented in the figure

An epistasis model establishes relationships among the phenotype expression frequencies for the different genotype combinations. Table 1 shows the additive model proposed in [10], where the odds increase multiplicatively with genotype both within and between loci. These relationships limit the possible prevalence (Eq. 1) and heritability (Eq. 2) combinations achievable by the model. Figure 2 represents the prevalence and the heritability as functions of the two variables from the second-order additive model shown in Table 1, using a MAF (Minor Allele Frequency) of 0.25 for both loci. This figure illustrates this limitation, as not every combination is present, e.g. there is no common point (*α*,*θ*) to both graphs where *P*(*D*)=0.8 and *h*^2^=0.2 and therefore it is not possible to reach both these values for the parameters using this model.

**Table 1:** Second-order additive model from [10], using the same genotypic effects for every loci

	BB	Bb	bb
AA	*α*	*α*(1+*θ*)	*α*(1+*θ*)^2^
Aa	*α*(1+*θ*)	*α*(1+*θ*)^2^	*α*(1+*θ*)^3^
aa	*α*(1+*θ*)^2^	*α*(1+*θ*)^3^	*α*(1+*θ*)^4^

Previous methods for calculating penetrance tables establish a desired prevalence and heritability and obtain the penetrance table as the solution to the system of equations formed by expressions (1) and (2) [14]. However, as not all combinations of heritability and prevalence are possible, these methods are prone to result in an incompatible equation system. Furthermore, since penetrances are probability values, they must be inside the interval [0,1]. Hence, the solution to the equation system needs to satisfy this condition as well.

To overcome these limitations, instead of finding a specific combination, the Toxo library maximizes one of the two parameters (prevalence or heritability) when the other is fixed. Once the maximum is calculated, the interval of achievable values is perfectly defined as the interval between 0 and the maximum. Following this approach, the likelihood of formulating an incompatible system when no information of the model is known is significantly reduced, since most of the models achieve all prevalences and heritabilities individually at some point. Toxo also considers the valid range of penetrance values as constraints to the equation system to be solved. Depending on the parameter to maximize (prevalence or heritability) the method slightly varies, so both will be explained in detail.

Taking into account Eq. 1, maximizing the prevalence means maximizing the sum:

3 $$ {\sum_{i}} \big(P(D|g_{i})P(g_{i})\big)  $$

where *P*(*D*|*g*_*i*_) is a function of the model variables (generally referred to as *x* and *y*) and *P*(*g*_*i*_) is constant for fixed MAFs, assuming Hardy-Weinberg equilibrium between the three genotypes at each locus and linkage equilibrium among the loci [14, 17]. To simplify the maximization process, we impose two restrictions to the input model:

1. All model expressions must be monotonically non-decreasing when *x* and *y* are real positive numbers.
2. The penetrance expressions must be sortable when *x* and *y* are real positive numbers.

These restrictions include the vast majority of models used in the literature, as will be discussed in “Model restrictions and existing epistasis models” section.

If the penetrance expressions are monotonically non-decreasing and sortable, all expressions will increment proportionally when increasing their variables. Consequently, their sum will reach its maximum value when the largest *P*(*D*|*g*_*i*_) expression also takes its maximum. Since penetrances are probabilities, their maximum value is 1. Therefore, we can obtain the maximum prevalence for a model, given a heritability value, by solving an equation system made of this heritability constraint and the condition of maximum prevalence:

4 $$ \begin{aligned} & \frac {{\sum_{i}} \big(P(D|g_{i}) - P(D)\big) P(g_{i})} {P(D)\big(1 - P(D)\big)} & & = h^{2} \\ & max\big(P(D|g_{i})\big) & & = 1 \end{aligned}   $$

The last step is to discard any solution with negative values for any of the variables of the model. The restrictions on the models are only true for real positive numbers and, as a result, there is no guarantee that negative solutions represent a maximum on the model.

An analogous process is followed to maximize the heritability when fixing the prevalence. On the heritability expression (Eq. 2), the only variable term is the sum in the numerator, since the prevalence and MAFs are fixed. Therefore, to maximize it we need to maximize the sum:

5 $$ {\sum_{i}} \big(P(D|g_{i}) - P(D)\big) P(g_{i})  $$

Using the same two restrictions as before, the sum will be maximum when the largest penetrance expression takes its maximum value since all expressions are monotonically non-decreasing. Again, we can obtain the maximum heritability for a model given its prevalence value by solving an equation system made of the prevalence expression and the condition of maximum heritability:

6 $$ \begin{aligned} & {\sum_{i}} \big(P(D|g_{i}) P(g_{i})\big) & & = P(D) \\ & max\big(P(D|g_{i})\big) & & = 1 \end{aligned}   $$

A complete numerical example of the method for the second-order additive model of Table 1 can be found in “Numerical example” section.

### Integration with other software

Toxo only calculates penetrance tables and it is intended to be used together with other software to complete the simulation of the data samples whose interactions correspond to those of the considered model. The design of Toxo is consequently focused on the integrability with third-party software. To accomplish this, Toxo relies on text files to communicate with other software.

An example of this integration is included with the source code [18] of the tool. In this case, GAMETES is used to simulate data using the penetrance tables generated by Toxo. The models are read by Toxo and its outputs (the calculated penetrance tables) are written following the GAMETES’ format. GAMETES then directly reads the file written by Toxo, a file comprised of all penetrances for the different allele combinations, and generates population samples using its own simulation method. Once it finishes, the result is a data file which segregates individuals as cases and controls, and for each individual the same genotype markers are specified.

Toxo offers complete flexibility on the output format of the table thanks to its object-oriented implementation, and it can be easily extended to support any other format required by a simulator.

## Results and discussion

### Model restrictions and existing epistasis models

As explained in “Calculating the penetrance tables” section, Toxo only admits models that meet two conditions:

- All model expressions are monotonically non-decreasing when the two model variables take real positive numbers.
- The penetrance expressions are sortable when the two penetrance variables take real positive numbers.

Nevertheless, these two conditions are met by several epistasis models that are currently actively used in the literature. These include Marchini’s second-order models [10] as well as their nth-order generalizations, the epistasis models proposed in experimental evaluation of BEAM [11], and the heterogeneity models introduced by Neuman and Rice [17].

The only example that we could find of a bivariate model that does not comply with the required conditions is Model 3 of [19], whose penetrance table is shown in Table 2. In this model the expression *α*/*f* is not monotonically increasing since it increases for *f*∈[0,1] and decreases for *f*∈[1,*∞*). Furthermore, the expressions of the model cannot be sorted for the real positive number space, as *α* is greater or equal than *α*/*f* for *f*∈[0,1] but lower for *f*∈(1,*∞*).

**Table 2:** Example of an incompatible model with Toxo, as shown in [19]

	BB	Bb	bb
AA	*α*	*α*	*α*
Aa	*αf*	*α*/*f*	*α*/*f*
aa	*αf*	*α*/*f*	*α*/*f*

Recent studies that include simulations based on epistasis models to generate their evaluation data [20–22] settle on low-order models whose heritability values are worryingly moderate. However, real-world diseases are usually determined by a higher number of genes [1] and a higher heritability [23, 24]. Our assumption is that previous works needed to use non-realistic low-order models and non-realistic heritability values due to limitations of state-of-the-art simulators, which are incapable of generating synthetic data with high heritability levels for high-order models. Toxo, together with current simulators, can facilitate current studies to overcome this limitation by finding appropriate penetrance tables and creating samples that resemble real-world data more closely.

### Numerical example

Assume that we work with the second-order additive model shown in Table 1. Our objective is to maximize the prevalence for a fixed MAF and heritability (in this example, 0.25 and 0.2, respectively). The first step consists in verifying that the model meets the specified criteria:

- Non-decreasing monotone expressions in the real positive number space: model expressions are monotonic in the real positive number space when its partial derivatives show no change in the sign for *x*>0 and *y*>0. The partial derivatives of all polynomial expressions for the second-order model are:
  $$\begin{array}{*{20}l} &\frac{\partial}{\partial x}\big(x\big) = 1 \\ &\frac{\partial}{\partial y}\big(x\big) = 0 \\ &\frac{\partial}{\partial x}\big(x(1+y)\big) = 1+y \\ &\frac{\partial}{\partial y}\big(x(1+y)\big) = x \\ &\frac{\partial}{\partial x}\big(x(1+y)^{2}\big) = (1+y)^{2} \end{array} $$
  $$\begin{array}{*{20}l} &\frac{\partial}{\partial y}\big(x(1+y)^{2}\big) = x(2y + 2) \\ &\frac{\partial}{\partial x}\big(x(1+y)^{3}\big) = (1+y)^{3} \\ &\frac{\partial}{\partial y}\big(x(1+y)^{3}\big) = x(3y^{2}+6y+3)\\ &\frac{\partial}{\partial x}\big(x(1+y)^{4}\big) = (1+y)^{4} \\ &\frac{\partial}{\partial y}\big(x(1+y)^{4}\big) = x(4y^{3} + 12y^{2} + 12y + 4) \end{array} $$
  All these derivatives are positive when *x*>0 and *y*>0.
- Sortable expressions in the real positive number space: all polynomial expressions can be sorted unequivocally:
  7) (8 $$\begin{array}{*{20}l} x \le x(1+y) \le x(1+y)^{2} \le x(1+y)^{3} \le x(1+y)^{4}, \\ \forall x,y \in \mathbb{R}, x,y \ge 0 \end{array} $$

After verifying that the model is appropriate for this method, the next step is to calculate the probability associated with each combination of two genotypes. Assuming linkage equilibrium between the two loci, and under the Hardy-Weinberg principle, the probability of a genotype can be calculated as the product of the probabilities of each allele [17]. This can be extended to any order of interaction by including the probabilities of each intervening allele in the product, provided that the same assumptions hold true. Thus, for an associated MAF of 0.25 for the two loci, the probabilities of each allele are $ p = \tfrac {1}{4} $ and $ q = 1 - p = \tfrac {3}{4} $, and the resulting allele combination probabilities are those shown in Table 3.

**Table 3:** Genotype probabilities of two loci combinations with the same MAF=0.25

	BB	Bb	bb
AA	$ \frac {81}{256} $	$ \frac {27}{128}$	$ \frac {9}{256} $
Aa	$ \frac {27}{128} $	$ \frac {9}{64} $	$ \frac {3}{128} $
aa	$ \frac {9}{256} $	$ \frac {3}{128} $	$ \frac {1}{256} $

Equations 4 have to be used in order to find the maximum prevalence for a fixed heritability value. The resulting equation system after replacing *P*(*D*|*g*_*i*_) with the model expressions from Table 1, and *max*(*P*(*D*|*g*_*i*_)) with the maximum expression, *x*(1+*y*)^4^, is:

9 $$ {\begin{aligned} & \frac{ {3xy^{2} \big(85y^{6} + 672y^{5} + 3264y^{4} + 9728y^{3}} {+ 19968y^{2} + 24576y + 16384\big)}} {{(y + 4)^{4} \big(256 - xy^{4} - 16xy^{3}} {- 96xy^{2} - 256xy - 256x\big)}} & & \!\!\,=\, & 0.2 \\ & x(1 + y)^{4} & & \!\!\,=\, & 1 \end{aligned}}  $$

The solution to the system, for *x*≥0 and *y*≥0, is *x*=0.0019 and *y*=3.7714. Table 4 shows the resulting penetrance table, which has an associated prevalence and heritability of 0.0275 and 0.2 respectively.

**Table 4:** Penetrance table of a second-order additive model with MAF=0.25, heritability=0.2 and maximum prevalence

	BB	Bb	bb
AA	0.0019	0.0092	0.0439
Aa	0.0092	0.0439	0.2096
aa	0.0439	0.2096	1

### Usage example

For the simple reason that Toxo is a programming library, it does not offer a graphical interface. Instead, it offers an API (Application Programming Interface) to its users so that any of its functions and methods can be used within any script or program. In order to describe the usage of Toxo, this section will exemplify how to generate a penetrance table for the second-order model of Table 1 with *MAF*=0.25 for both loci that can be loaded directly into GAMETES [12] to generate data samples.

The first step to create a penetrance table is to define the epistasis model to be used. It must be written to a file using CSV (Comma-Separated Values) format, where rows correspond to the different genotypes and two columns define the genotype and its associated penetrance expression. The two variables are arbitrarily named *x* and *y* (Toxo interprets any alphabetic characters in the penetrance expressions column as variable names). To define the second-order additive model, a file named model.csv is created containing the following information: 
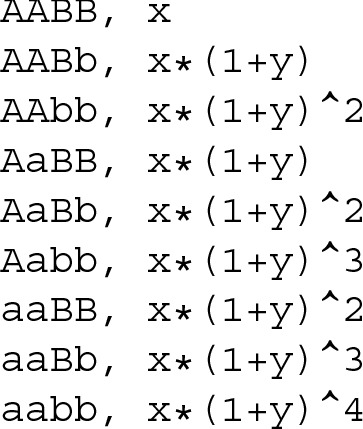


Once the model file is created, an instance of the class Model can be created by reading it: 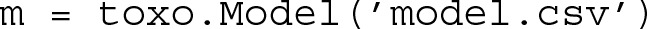


From this Model instance, the penetrance table with maximum prevalence can be found using the method find_max_prevalence. The parameters of this method are the MAF for each of the two loci of the model given as a vector and the heritability constraint. Following the example, the function call to create a penetrance table for the model with MAFs 0.25 and target heritability 0.2 is: 
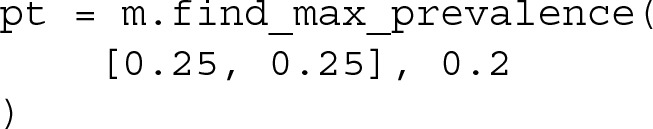


In the case of looking for the table with maximum heritability, the method to be called instead is find_max_heritability. The parameters of this method are, again, the MAF for each of the two locus of the model given as a vector and the prevalence constraint of 0.1 instead of the heritability: 
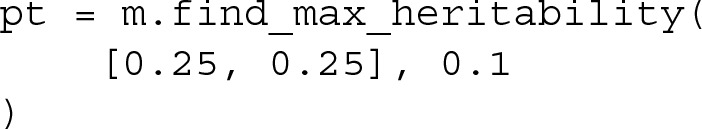


Finally, the calculated penetrance table can be written to a file so that a simulator can make use of it to generate data sets, which can be done using the method write of the class PTable. The output format is chosen using different constants statically declared inside class PTable. In our example, to use GAMETES we have to introduce the format_gametes constant: 
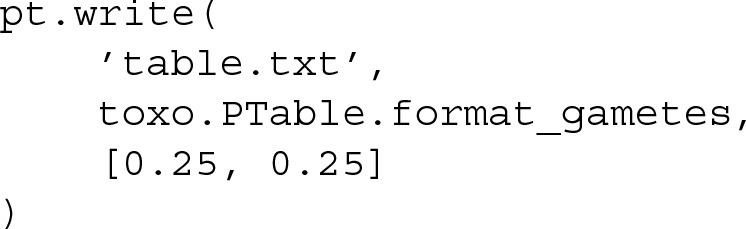


The resulting file table.txt can be loaded as a model inside GAMETES, and data can be simulated from it. The code included in this example is also available at the Github repository [18], which can be executed line by line to further comprehend the usage of Toxo.

### Evaluation

Evaluation of Toxo focuses on two different aspects of the library: the precision of the results and the runtime. All the tests were run on a 64-bit Linux machine with two eight-core Intel Xeon E5-2660 CPUs and 64 GB of RAM, using the command line interface of MATLAB version R2018a (9.4.0.813654).

A battery of tests was developed to evaluate the precision of the results (the difference between the requested and the observed heritability) and the runtime. All executions were repeated five times and their runtimes averaged to avoid outliers. Table 5 shows the results for the additive, multiplicative and threshold models [10], generalized for third and fourth-order, and for a variety of MAF and heritability values. The evaluation is focused on the heritability since it is the parameter with the most interest in case-control studies, whereas the prevalence is not as important because having a fixed number of cases and controls negates the effect of phenotype frequency in a non-controlled environment. The selection of models ranges from a very simple model like the threshold (where all the polynomials inside the model are of first degree) to a more complex one like the multiplicative (where the degree is generally higher). The MAF and heritability combinations were also chosen to show a wide spectrum of values. Results show that the precision error is almost nonexistent for every test. As for the runtimes, all the tables were able to be calculated in under a quarter of a minute, with the only exception being the fourth-order multiplicative model, which took a little more than two minutes.

**Table 5:** Precision error of the heritability obtained for the penetrance table and execution time, calculated under several model, MAF and heritability configurations

Model	Order	MAF	*h*^2^	Error	Time (s)
Additive	3	0.1	0.1	0	7.06
Additive	3	0.1	0.8	1.31E-05	7.08
Additive	3	0.4	0.1	0	6.89
Additive	3	0.4	0.8	9.99E-16	6.95
Additive	4	0.1	0.1	1.58E-12	14.17
Additive	4	0.1	0.8	4.04E-12	13.14
Additive	4	0.4	0.1	0	13.59
Additive	4	0.4	0.8	3.92E-03	13.61
Multiplicative	3	0.1	0.1	0	8.60
Multiplicative	3	0.1	0.8	0	8.51
Multiplicative	3	0.4	0.1	0	8.03
Multiplicative	3	0.4	0.8	0	7.82
Multiplicative	4	0.1	0.1	0	142.32
Multiplicative	4	0.1	0.8	0	145.94
Multiplicative	4	0.4	0.1	0	90.05
Multiplicative	4	0.4	0.8	0	85.42
Threshold	3	0.1	0.1	0	2.55
Threshold	3	0.1	0.8	0	2.54
Threshold	3	0.4	0.1	0	2.50
Threshold	3	0.4	0.8	0	2.50
Threshold	4	0.1	0.1	0	3.57
Threshold	4	0.1	0.8	0	3.57
Threshold	4	0.4	0.1	0	3.59
Threshold	4	0.4	0.8	0	3.58

To compare these results with state-of-the-art competitors, the same table configurations were attempted in EpiSIM [14]. Although gs [15] can also calculate penetrance tables from epistasis models containing bivariate functions, it is not included in the comparison as it does not allow modifying the second-order embedded models included within the program. EpiSIM, on the other hand, requires both the prevalence and heritability to obtain a penetrance table. To make a fair comparison, two different cases were tested for each of the configurations defined: one with the exact same prevalence and heritability combination obtained by Toxo, and a second one with the former heritability and a fixed prevalence value (1E-20), supposedly easier to find since it is below the maximum. Despite this, EpiSIM could not find a single table for any of the tests.

## Conclusions

The main contribution of this work is the creation of a library, Toxo, capable of calculating penetrance tables from models containing bivariate penetrance functions with no limitations on the interaction order. It allows the user to maximize the prevalence of the resulting table when the heritability is constrained and vice versa. In addition, Toxo can be easily integrated with other existing simulators to generate data sets that include the epistasis relationships described in the penetrance table.

Thanks to the mathematical method used underneath, Toxo can calculate penetrance tables with prevalence and heritability values much higher than those observed in the state of the art. The majority, if not all, of the works in the literature use heritabilities under 0.2 for high-order penetrance tables. However, it is believed that real world diseases present higher heritabilities. Toxo provides researchers with a library to generate penetrance tables and, in consequence, data samples that resemble characteristics from real world diseases more closely.

Empirical results show that Toxo is capable of calculating penetrance tables for high-order models according to the specified parameters with barely any precision error. Third-order tables can be obtained in under 10 seconds, and fourth-order tables in about 2 minutes.

The current implementation, however, also comes with its own limitations. The maximum interaction order that Toxo can handle is determined by MALTAB equation solvers. When using polynomials of sufficient degree, MATLAB is unable to solve the proposed equation. For example, using the additive model, the implementation can obtain penetrance tables of up to 10th order. Ease of use is also an aspect that can be improved. Users unfamiliar with command-line interfaces or with little programming background may find Toxo difficult to use. Output formats for the penetrance tables are also limited, currently only supporting GAMETES format natively.

Future work will be focused on improving Toxo usability following two lines: natively supporting a larger number of output formats, and providing Toxo with a graphical interface. These changes aim to simplify its usage, allowing Toxo to reach a larger community of users.

## Availability and requirements

Project name: Toxo Project home page: https://github.com/chponte/toxoOperating system(s): Platform independent Programming language: MATLAB Other requirements: None License: MIT Any restrictions to use by non-academics: None

## Data Availability

The source code of Toxo, usage instructions and all the models and code examples used within this paper, are available in the Github repository: https://github.com/chponte/toxo.

## Abbreviations

GWAS: Genome-Wide Association Study
MAF: Minor Allele Frequency
API: Application Programming Interface
CSV: Comma-Separated Values
